# Predictors of chest drainage of pneumothorax in neonates

**DOI:** 10.1590/1414-431X20209469

**Published:** 2020-06-26

**Authors:** Ya-lan Tan, Yang Zhan, Jia Geng, Wei Chen, Wan-Liang Guo

**Affiliations:** 1 Department of Radiology, Children's Hospital of Soochow University, Suzhou, China; 2 Department of Radiology, The First Affiliated Hospital of Soochow University, Suzhou, China; 3 Clinical Laboratory, 3rd Hospital of Yulin City, Yulin, China

**Keywords:** Neonate, Pneumothorax, Chest drainage, Predictor

## Abstract

This is a retrospective, single-center observational study to explore the predictors of chest drainage for neonatal pneumothorax. A total of 183 neonates (age ≤28 days) who presented to the Children's Hospital of Soochow University between January 1, 2015 and December 31, 2018 for pneumothorax or developed pneumothorax during a hospital stay were included. Demographic data, clinical presentation, and imaging characteristics of neonatal pneumothorax were collected and analyzed. We used univariate and multivariate logistic regression analyses to determine significant predictors of chest drainage of pneumothorax in neonates. Pneumothorax occurred within 24 h after birth in 131 (71.6%) cases, between 24 and 48 h after birth in 41 (22.4%) cases, and 48 h after birth in 11 (6.0%) cases. Univariate and multivariate logistic regression analyses revealed that lung collapse ≥1/3 on initial chest X-ray (OR 4.99, 95%CI 2.25-11.07), chest retractions (OR 8.12, 95%CI 2.88-22.89), cyanosis (OR 2.25, 95%CI 1.08-4.66), and frothing from mouth (OR 2.49, 95%CI 1.12-5.49) (P<0.05 for all) were significant predictors of the need for chest drainage due to pneumothorax. In conclusion, the thorough evaluation of the above predictive factors can guide treatment and improve patient outcome.

## Introduction

The incidence of neonatal pneumothorax is 1–2% in the general population, and could be as high as 6–10% in infants with a birth weight of ≤1,500 g, with a male predominance (1,2). In most cases, neonatal pneumothorax occurs within 3 days after birth. Large-tension pneumothorax could increase intrathoracic pressure and decrease cardiac output. Failure to follow the proper treatment approach for pneumothorax in neonates will lead to serious complications, including lung perforation, phrenic nerve palsy, chylothorax, and hemopericardium. However, the early and effective management of pneumothorax in neonates is challenging to pediatricians and pediatric surgeons.

Previous studies have noted that the rate of chest drainage was 59% and factors potentially influencing the need for drainage were nasal continuous positive airway pressure treatment, oxygen treatment, maximal oxygen percentage, and mechanical ventilation (3). Similarly, Al Matary et al. (4) reported 86 neonatal pneumothorax, and concluded that needle aspiration, chest tube insertion, and ventilator support should be considered if the patient presents with respiratory distress and poor blood gas changes. However, Kitsommart et al. (5) reported a case series in which massive pneumothorax with mediastinal shift was successfully managed without chest drainage. To date, there are few studies on the predictors of chest drainage for pneumothorax in neonates. In addition, sample sizes were relatively small and most studies used univariate analysis; thus, indications for chest drainage require further studies.

Unlike the previous studies, we analyzed a relative large sample of neonatal pneumothorax, and multivariate logistic regression modeling was conducted to identify the predictors of chest drainage in neonatal pneumothorax.

## Material and Methods

### Study subjects

Medical records of neonates (age ≤28 d) receiving treatment as in-patients at the Children's Hospital of Soochow University during the 4-year period from January 1, 2015 to December 31, 2018 were systematically searched to identify cases of neonatal pneumothorax. The cases included those who presented with pneumothorax upon hospital admission as well as those who later developed pneumothorax during hospital stay. The diagnosis was confirmed by chest X-ray as described previously (6).

Neonates with pneumothorax, if asymptomatic, were observed conservatively. For neonates with mild symptoms, good general condition, and small pneumothorax, conservative treatment (proper sedation, oxygen inhalation, anti-infection therapy) was adopted. The chest tube insertion was performed by the pediatricians as soon as the clinical indication (presence of respiratory distress, abnormal blood gas levels, and cardiovascular instability) was evident.

The study protocol was approved by the Institutional Ethics Review Committee at Children's Hospital of Soochow University.

### Factor analysis

Demographic data, clinical presentation, and imaging characteristics were analyzed for the two groups using univariate and multivariate analyses.

Caesarean section (C-section) was defined as the birth of a fetus through a surgical incision on the abdominal wall (laparotomy) and uterine wall (hysterotomy) (7). Meconium-stained amniotic fluid was defined as the passage of fetal colonic contents into the amniotic cavity during pregnancy due to different reasons, which could cause adverse long- and short-term fetal outcomes involving increased rates of neonatal resuscitation, respiratory distress, lower Apgar score, neonatal nursery admissions, meconium aspiration syndrome, neonatal sepsis, and pulmonary disease (8). Nuchal cord was defined as the umbilical cord being wrapped 360 degrees around the fetal neck at least once (9). Placental abruption was defined as the premature separation of the placenta before delivery (10). Perinatal asphyxia and resuscitation, also called birth asphyxia, was defined as the failure to initiate and sustain breathing at birth. If newborns have asphyxia, some assistance at birth and more extensive resuscitative measures like ventilation and chest compression are performed to restore breathing and improve circulatory function (11).

Groan was defined as a deep inarticulate sound conveying pain. Frothing from the mouth was defined as a rising or overflowing mass of small bubbles from the mouth. Chest retractions was defined as suprasternal, intercostal, or subcostal retractions. Cyanosis was defined as a bluish-purple discoloration of the skin and mucous membranes resulting from a deficiency of oxygen in the blood.

Meconium aspiration syndrome was defined as respiratory distress in meconium-stained infants within 12 h of age, displaying symptoms such as hypoxia, tachypnea, gasping respiration, and signs of underlying asphyxia, with a chest radiograph showing overexpansion of the lungs with widespread coarse infiltrates (12). Respiratory distress syndrome was defined by the following criteria: respiratory difficulty (i.e., tachypnea, grunting, retractions, and cyanosis), persistent oxygen requirement over the first 48 to 96 h of life, a diffuse ground-glass appearance with air bronchograms and hypoexpansion on chest radiography, and hypoxemia and acidosis on blood gas analysis (13). Neonatal pneumonia was defined by the following clinical criteria: infants presenting with increased work of breathing and oxygen requirement, and diffuse parenchymal infiltrates with air bronchograms or lobar consolidation demonstrated on chest radiography (14). Congenital heart disease in this study referred to infants with atrial septal defect, ventricular septal defect, or patent ductus arteriosus without surgical treatment.

### Statistical analysis

All analyses were conducted using the SAS software V.9.2 (SAS Institute, USA). Continuous variables following normal distribution are reported as means±SD, and were analyzed using Student's *t*-test for between-group comparisons. Continuous variables not following normal distribution are reported as median (P_25_-P_75_), and analyzed using Wilcoxon rank sum test. Categorical variables are reported as number (rate), and analyzed using chi-squared test, continuity correction chi-squared test, or Fisher exact probability method for between-group comparisons. Variables with significant between-group differences (P<0.05) were entered into the multivariate logistic regression analysis to identify factors associated with chest drainage. The receiver operating characteristic curve (ROC) and the Hosmer-Lemeshow goodness-of-fit test were used to assess the performance of derived models. P<0.05 (2-sided) was considered statistically significant.

## Results

### Demographic and clinical characteristics of the study population

**Figure 1:**
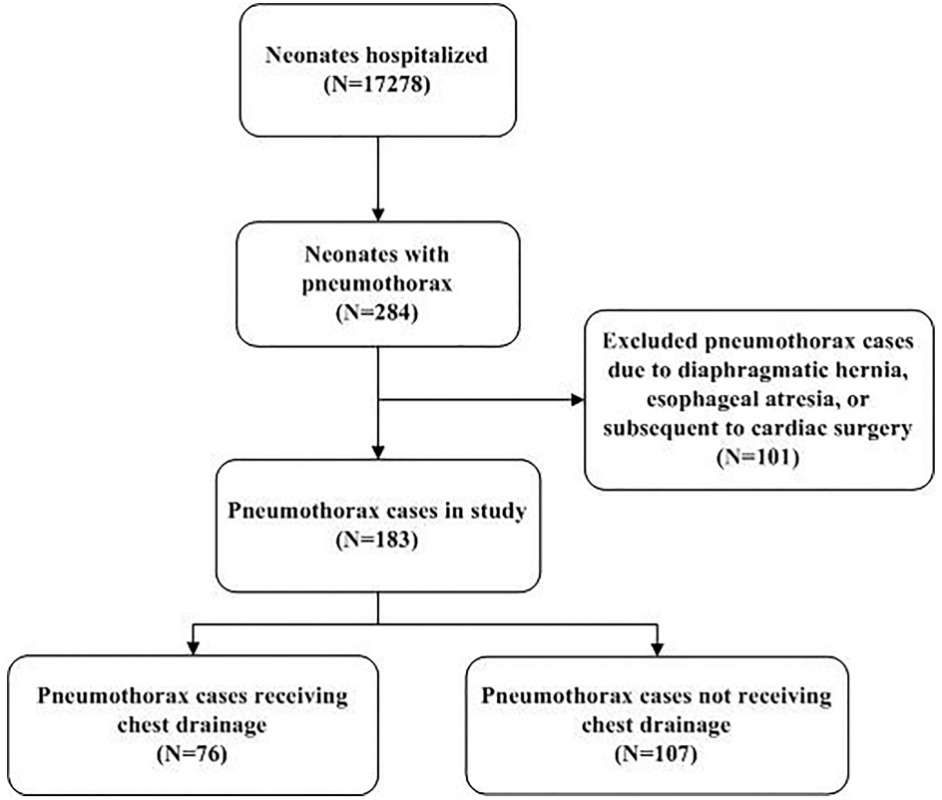
Flow chart of case selection.

We screened 17,278 hospitalized neonates and a total of 183 neonates with pneumothorax met the study inclusion criteria (Figure 1). The demographic and clinical characteristics of participants are shown in Table 1. Pneumothorax occurred within 24 h after birth in 131 (71.6%) cases, between 24 and 48 h after of birth in 41 (22.4%) cases, and 48 h after birth in 11 (6.0%) cases.

**Table 1:** Demographic and clinical characteristics of neonates admitted with pneumothorax (n=183).

Variables	n (%)
Demographic data	
Gender, male	114 (62.3)
Gestational age,<37 weeks	72 (39.3)
Birth weight, <2500 g	44 (24.0)
Perinatal data	
C-section	122 (66.7)
Meconium-stained amniotic fluid	41 (22.4)
Nuchal cord	16 (8.7)
Placental abruption	12 (6.6)
Perinatal asphyxia and resuscitation	38 (20.8)
Neonatal data	
Meconium aspiration syndrome	11 (6.0)
Respiratory distress syndrome	31 (16.9)
Pneumonia	141 (77.1)
Congenital heart disease	29 (15.8)
Mechanical ventilation before pneumothorax	92 (50.3)
Mortality	2 (1.1)

### Chest drainage

Among the 183 neonates included in data analysis, 76 received chest drainage and the remaining 107 did not receive drainage.

The subjects that received chest drainage *vs* those who did not differed significantly in rates of C-section, perinatal asphyxia resuscitation, a history of mechanical ventilation prior to pneumothorax, tachypnea, groan, frothing from mouth, chest retractions, enlarged hemithorax on the involved side, cyanosis, neutrophil ratio, C-reactive protein, and degree of lung collapse (P<0.05) (Table 2).

**Table 2:** Clinical and imaging characteristics in neonates with pneumothorax receiving or not chest drainage.

	Drainage (n=76)	No drainage (n=107)	P
Demographic data			
Gender, male	27 (35.5)	42 (39.3)	0.6083^a^
Gestational age, <37 weeks	36 (47.4)	36 (33.6)	0.0611^a^
Birth weight, <2500 g	16 (21.1)	28 (26.2)	0.4249^a^
Perinatal data			
C-section	58 (76.3)	64 (59.8)	0.0196^a^
Meconium-stained amniotic fluid	12 (15.8)	29 (27.1)	0.0705^a^
Nuchal cord	5 (6.6)	11 (10.3)	0.3824^a^
Placental abruption	7 (9.2)	5 (4.7)	0.3581^b^
Perinatal asphyxia and resuscitation	10 (13.2)	28 (26.2)	0.0325^a^
Neonatal data			
Meconium aspiration syndrome	3 (3.9)	8 (7.5)	0.5002^b^
Respiratory distress syndrome	15 (19.7)	16 (15.0)	0.3953^a^
Pneumonia	58 (76.3)	83 (77.6)	0.8424^b^
Congenital heart disease	16 (21.1)	13 (12.1)	0.1041^a^
Mechanical ventilation before pneumothorax	45 (59.2)	47 (43.9)	0.0416^a^
Clinical manifestations at diagnosis			
Tachypnea (>60/min)	75 (98.7)	81 (75.7)	<0.0001^a^
Heart rate (bpm)	140 (131-145.5)	140 (130-145)	0.4234^c^
Groan	27 (35.5)	22 (20.6)	0.0243^a^
Frothing from mouth	32 (42.1)	29 (27.1)	0.0339^a^
Chest retractions	71 (93.4)	56 (52.3)	<0.0001^a^
Enlarged hemithorax on the involved side	14 (18.4)	8 (7.5)	0.0249^a^
Cyanosis	42 (55.3)	26 (24.3)	<0.0001^a^
Laboratory examination at diagnosis			
Leukocyte count (×10^9^/L)	16.02 (13.03-20.92)	17.44 (13.30-22.57)	0.2547^c^
Neutrophil ratio (%)	75.75 (69.30-80.50)	71.5 (62.7-76.9)	0.0065^c^
C-reactive protein (mg/L)	2.105 (0.645-8.135)	0.67 (0.26-2.80)	0.0002^c^
First chest X-ray manifestation			
Bilateral pneumothorax	11 (14.5)	14 (13.1)	0.7874^a^
Lung collapse ≥1/3	41 (53.9)	21 (19.6)	<0.0001^a^
Pneumomediastinum	11 (14.5)	12 (11.2)	0.5123^a^

Categorical variables are reported as number (%). Continuous variables are reported as median (interquartile range, 25–75).

^a^Chi-squared test; ^b^Continuity correction chi-squared; ^c^Wilcoxon rank sum test.

**Figure 2:**
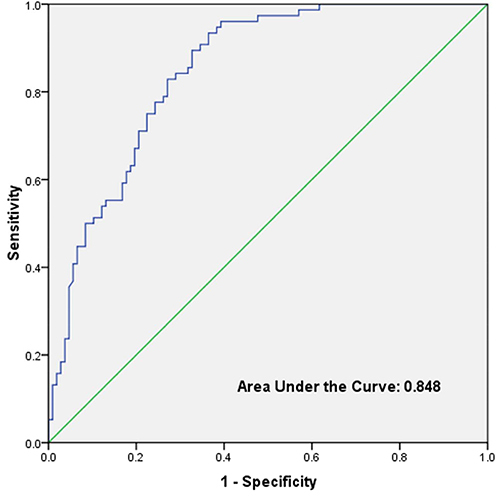
Receiver operating characteristics curves for the prediction model of pneumothorax drainage in neonatal pneumothorax (AUC=0.848).

Multivariate analysis revealed the following predictors for chest drainage: chest retractions, frothing from mouth, cyanosis, and lung collapse ≥1/3 (P<0.05) (Table 3). The logistic regression model agreed with the Hosmer-Lemeshow's goodness-of-fit test (P=0.4370). The area under the ROC curve analysis was 0.848 (Figure 2).

**Table 3:** Predictors of chest drainage in neonates with pneumothorax.

	Univariate regression			Multivariate analysis^a^		
	OR	95%CI	P	OR	95%CI	P
C-section	2.16	1.12-4.17	0.0209			
Perinatal asphyxia and resuscitation	0.43	0.19-0.94	0.0356			
Mechanical ventilation before pneumothorax	1.85	1.02-3.36	0.0425			
Tachypnea (>60/min)	24.07	3.19-181.81	0.0020			
Groan	2.13	1.10-4.13	0.0257			
Enlarged hemithorax on the involved side	2.79	1.11-7.05	0.0294			
Neutrophil ratio (%)	1.03	1.00-1.06	0.0230			
C-reactive protein (mg/L)	1.05	1.01-1.10	0.0225			
Chest retractions	12.93	4.84-34.56	<0.0001	8.12	2.88-22.89	<0.0001
Lung collapse ≥1/3	5.10	2.62-9.89	<0.0001	4.99	2.25-11.07	<0.0001
Frothing from mouth	1.96	1.05-3.65	0.0350	2.49	1.12-5.49	0.0245
Cyanosis	3.85	2.05-7.24	<0.0001	2.25	1.08-4.66	0.0298

^a^Forward multivariate regression model was used in this multivariate analysis.

Hosmer-Lemeshow’s goodness-of-fit test (χ^2^=6.9221; P=0.4370).

## Discussion

In this study, we analyzed a series of clinical and imaging data of neonatal pneumothorax and multivariate logistic regression was conducted to identify the predictors of chest drainage in neonatal pneumothorax. One of the findings in the current study was the independent association of need for chest drainage with lung collapse ≥1/3 on initial chest X-ray, chest retractions, and cyanosis in neonates. The findings were consistent with studies in adult patients with pneumothorax (15).

Esra et al. (16) reported that newborns with a pneumothorax size greater than 20% are likely to have a worse prognosis and risk of mortality 13 times higher than those with smaller pneumothorax size. It indicated that more aggressive treatments like drainage may be warranted in large pneumothorax. By comparing patients with and without chest tube insertion, Esra et al. also found that pneumothorax size is significantly higher in neonates who were treated with a chest tube (25.8±1.9% *vs* 14.2±2.0%, P<0.001), indicating that pneumothorax size may be an influencing factor for drainage treatment. The current study result was similar to the above, and further confirmed that lung collapse ≥1/3 on initial chest X-ray was an independent predictor of chest drainage by using multivariate analysis.

The present study also showed an association between the need for chest drainage with frothing from the mouth. In our opinion, frothing from the mouth most likely reflected pneumonia and severe lung pathologies that are not conducive to pneumothorax absorption. Pro-active intervention is necessary. Vibede et al. (3) compared 42 neonates that received chest drainage for pneumothorax and 39 neonates that did not and found that neonates with underlying lung diseases are more likely to require chest tube placement.

There were several limitations in the current study. First, this was a retrospective study, thus selection bias could have affected data interpretation. For example, asymptomatic neonatal pneumothorax might have been missed. CPR/hemodynamic instability is one variable that might affect chest tube placement as well as mortality. Also, the conclusions were based on data from a single center so a multicenter study is needed to further evaluate the predictors of chest drainage for pneumothorax in neonates.

In conclusion, this retrospective analysis confirmed the independent association of the need for chest drainage with lung collapse ≥1/3 on initial chest X-ray, chest retractions, cyanosis, and frothing from the mouth. Whether the findings could be generalized to general medical practice requires future studies.
